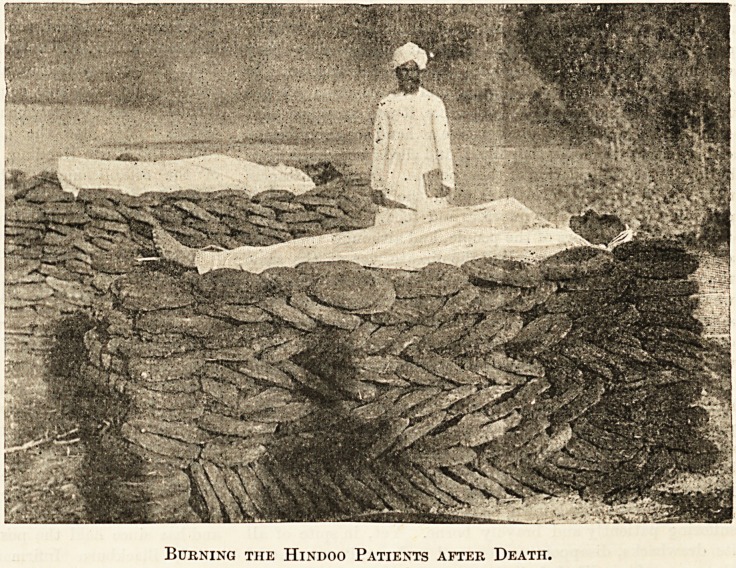# "The Hospital" Nursing Mirror

**Published:** 1899-07-29

**Authors:** 


"The Hospital" Nursing Mirror.

Being the Nursing Section of "The Hospital."

[Contributions for this Section of "The Hospital" should be addressed to the Editor, The Hospital, 28 & 29, Southampton Street, Strand, London, W.C., and should have the word "Nursing" plainly written in left-hand top corner of the envelope.]

### Notes on News from the Nursing World.

### THE NURSING EVENT OF THE YEAR.

In another part of the "Nursing Mirror" will be found full and descriptive reports of the great event of Friday last—the reception by the Prince and Princess of Wales of some twelve hundred nurses, and the presentation to them by the Princess of Wales, in her capacity of President of the Royal National Pension Fund, of their certificates. The brilliance of the function was enhanced by the glorious weather, and its enjoyment was heightened for all by the admirable manner in which everything was managed. That the nurses themselves heartily appreciated the privilege of being received and entertained by their President and the Prince was clear enough from their radiant faces; but we have since received several letters from those who were present expressing their gratification. For example, the lady superintendent of an important nursing home in the provinces, who was herself one of the first thousand enrolled as members of the Fund, writes that her nurses will "never forget the day, which everybody concerned took so much trouble to make a success." On the other hand the nurses themselves, by their implicit obedience to instructions, and by the enthusiasm they manifested at all stages of the proceedings, materially contributed to the effectiveness of the ceremony. One word remains to be said about the Royal National Pension Fund itself. In the graceful reply addressed by the Prince on behalf of the Princess to Sir Henry Burdett, His Royal Highness observed "that there is no philanthropic object with which we could be more gladly associated than this Pension Fund for Nurses." This is most encouraging to everyone associated with the movement, and cannot fail to have the practical result of both increasing the interest taken in the Fund, and of adding to the number of policyholders. We may repeat that the nurses who were unable to attend the ceremony can have their certificates sent to them by post on application by letter to the office of the Fund, enclosing a penny stamp, and quoting their policy number.

### THE PENSION FUND OFFICE.

There were few at Marlborough House on Friday accustomed to witness ceremonials who were not surprised at the excellent organisation apparent from first to last. The result was due to the hearty co-operation of all concerned: The officials at Marlborough House, General Crease and his sergeants, the members of the police force, the nurses themselves, and last and not least to the officials of the Pension Fund, and it is of these latter we desire to record a few words of appreciation. The notice of the intended ceremony was exceedingly short, but ten days were available for the issue of nearly 3,000 invitations and directions, the provision of apartments for those desiring to remain in London the previous night, the railway arrangements, the printing of certificates, and the organisation of the whole ceremony. Now the staff at the Pension Fund is as small as possible to carry on the usual work, and daily life there at the office is a busy one. It can be imagined that such a ceremony as that of Friday would tax the resources of Mr. Dick and his assistants. Yet so anxious were all concerned that no hitch should occur that all put their shoulders to the wheel, worked some thirteen hours a day, and only added two to the usual number of the staff, though extra premises had to be engaged to carry out the arrangements. We are glad to learn that services so heartily and spontaneously given have been much appreciated. Mr. Dick has been really touched and gratified by the many kind letters he has received from nurses, and General Crease commented upon the able way in which all the work of himself and his sergeants had been facilitated by the admirable manner in which all had been prepared. In the few days before the ceremony no less than 2,430 letters poured into the office, apart from the usual correspondence. This will enable those who have not already done so to realise the trouble and forethought which was exercised by those connected with the Pension Fund.

### THE LATE CHAIRMAN OF THE PENSION FUND.

Nurses of the Royal National Pension Fund will be glad to hear that friends of the late Mr. Walter Burns, who was chairman of the Fund since its foundation until his death, have endowed a cot at Guy's Hospital to perpetuate his memory. Guy's Hospital Nurse Training School is affiliated with the Pension Fund, and the matron is one of the nurses' representatives who administer the Junius S. Morgan Benevolent Fund. The event will therefore awaken special interest at Guy's.

### THE "MARY ADELAIDE" NURSES.

After the reception of the nurses of the Royal National Pension Fund, on Friday, the "Mary Adelaide" nurses—*i.e.*, the nurses belonging to the Workhouse Infirmary Nursing Association—who were present at the ceremony, were entertained at tea by Lady Wantage, at 2, Carlton Gardens. The distribution of the medals and gratuities to the nurses who were entitled to them this year took place at the same time and, in fact, this usual annual gathering, generally held in May, was deferred in order to fit it in with the date chosen by the Princess of Wales for the reception at Marlborough House.

### THE CARE OF THE SICK IN OUR COLONIES.

At the annual meeting of the Colonial Nursing Association, on Tuesday, Mr. Chamberlain, with his usual felicity, contrived to invest the topic of nursing in the Colonies with fresh interest. For the sake of lives precious to the Empire, he called upon the public to afford the Association the modest financial support which it still requires. The Colonial Secretary recognises the fact that in tropical climates good nursing may be successful even after the most skilful medical man has exhausted his resources. It is no figure of speech to say that patients given up by doctors have been nursed back to life again; and if for no other reason than this the Association, whose claims Mr. Asquith, as well as Mr. Chamberlain, pleaded so forcibly, should not for a moment be embarrassed for want of funds. In this case the labourers are many and capable, though the work is trying and dangerous. The only need is a sum of £3,000 to make up a guarantee fund of £5,000. This comparative trifle a generous and discerning public will, we are convinced, promptly subscribe. They will not forget that the obligations of Empire include the obligation of sending out nurses to its dependencies in all parts of the world.

### THE NEW SOUTH WALES ARMY NURSING SERVICE RESERVE.

It will be remembered that in October last we mentioned that the Government authorities of New South Wales had sanctioned the formation of an Army Nursing Service Reserve. Colonel Williams, who commands the New South Wales medical services, writes us from Sydney that the formation of the service has been completed, and that Miss Ellen Julia Gould, member of the Royal British Nurses' Association, has been gazetted as lady superintendent of nurses, and Miss Julia Bligh Johnstone as superintendent.

### "ANGELS OF THE HOSPITAL."

Under this title the *St. Louis Globe Democrat* has an article on the long and severe training to which nurses are subjected "before they are considered adepts in their profession." It is certain that they usually, and with much sense, aspire to be adepts rather than angels, but no doubt other things being equal the tenderness of their ministrations enhances the value of them. Our American contemporary having dwelt at length on the manner in which nurses are trained, winds up with the startling statement that, though the profession is not considered as specially unhealthy, a hospital superintendent in St. Louis says that "the average life a nurse is but ten years." Whatever may be the experience in respect to America, or any portion of it, this is not the case in Great Britain. In fact, we are very much afraid that if entering the nursing profession meant an average life of ten years only, the number of "Angels of the Hospital" would be seriously diminished. But if the superintendent of the St. Louis Hospital had been at the Reception at Marlborough House on Friday, he would have come to the conclusion that nursing in England by no means implies a short life.

### NURSING IN THE RURAL DISTRICTS.

At a very large meeting in Luton Town Hall, held for the purpose of showing the need for extending the work of the Bedfordshire Rural Nursing Association, Mr. W. W. Marks cited some extraordinary instances of the ignorance of the rural poor on the subject of sickness. One was that of a woman who, when her husband fell sick, was told to keep the invalid in a recumbent position. "The poor woman," continued Mr. Marks, "never having heard of the word 'recumbent' before, went to her neighbour and asked if she had one. The neighbour said she had not, but probably if she went to the grocer he would give her one." Such examples as this may well be put forward in support of the plea for the employment of proper nurses in the rural districts. Mr. Samuel Whitbread states that the Bedfordshire Association has "to content itself with partially-trained nurses." But it must be admitted that he has a reason. "Fully-trained nurses," he affirms, "are not to be had; the demand exceeds the supply." The partially-trained nurses, of course, go through six months' training in one of the large London hospitals, and have to pass an examination of the Obstetrical Society for a certificate.

### THE GRIMSBY NURSING INSTITUTION.

Miss Garrett, who was lady superintendent during the cholera and typhoid epidemics at Grimsby, writes: "One of my happiest experiences was at the Grimsby Nursing Institution. Much to my regret, urgent private reasons compelled me to resign such an interesting sphere of work. In my varied experience I never worked with a more courteous, appreciative committee. I always found a valuable official friend in the lady who has so kindly acted as hon. secretary almost ever since the institution was started in 1890; her tact and good judgment were an immense help to me. Had things fitted in, I should have esteemed it a privilege to have returned and again worked as superintendent of this well-organised institution, which deservedly holds a foremost place in the town of Grimsby. Without entering into details, it is only fair to state that any change of matron that has occurred has most certainly not been from any fault of the committee."

### THE NATIONAL ORTHOPÆDIC HOSPITAL.

It was decided some time ago to rebuild the old part of the National Orthopædic Hospital, in Great Portland Street. This will be an enormous gain to the nurses, as at present the accommodation provided for them is very inadequate; in fact, there are no proper quarters at all. The deficiency, however, will be fully remedied when the new buildings are completed. The president, the Duke of Marlborough, and his wife, take a personal interest in the patients, and visit the hospital constantly. The Ladies Committee also show an active oversight in their welfare. Amongst other things they organise an annual sale of work, the proceeds of which augment the Extra Comforts Fund. This year amongst the purchases are new linen cupboards, a further supply of the handsome oak lockers that are gradually superseding the shabby ones now in use, as well as some new beds. The work, especially amongst the children, is interesting, and promising young candidates are glad to receive their initiation into the nurses' career under the highly-trained sisters who take charge of the wards. Two years' training is given before the probationer is passed on to a general hospital; but there is rarely a vacancy.

### THE LATE NURSE FRANCES EDWARDS.

A Yorkshire nurse sends us the following: "Reading in The Hospital the account of the death of Nurse Frances Edwards, I thought the public would like to know a little about her, for I am sure she is well known to many in the profession. She was a bright, attractive girl when I first knew her, and was a first assistant nurse at one of the London fever hospitals. During her stay there one night, going from her room back to the ward, it being dark, she struck her head a tremendous blow against one of the corridor pillars, which rendered her unconscious and seriously ill for several days. After her recovery (her illness lasted several weeks) she frequently complained of intense headache, and used to get fits of depression which I imagined she had never recovered from, and her asylum experience would not by any means help or improve her. Her father is a clergyman in some part of Australia. She came over to England after having received her training at Prince Alfred's Hospital. Under these sad circumstances I am sure the friends of the deceased have the fullest sympathy of the nursing profession."

### THE NURSES' TOTAL ABSTINENCE LEAGUE.

A delightful "At Home" was given by Miss Hilda E. Dillon at her father's house, 47, Oakley Street, to the members of the Nurses' Total Abstinence League on Friday evening. Miss Dillon selected this graceful mode of assuming the office of honorary secretary to the League, in succession to the late much-valued secretary, Miss Holland. A large number of guests accepted the invitation, and were received by the President, the Hon. Mrs. Elliot Yorke, who subsequently spoke most earnestly to them, entreating them to do all in their power to carry on the work of the League. Miss S. Ellen Orme, in a short address, urged the members to help others to do the best they could; and the Hon. Conrad Dillon, in the absence of his wife, bade his daughter's guests welcome, and wished success to the League. Then, for the benefit of the strangers present, Mrs. Finlay explained the work of the organisation, coffee and other refreshments being afterwards served in the garden.

### DELHI MEDICAL MISSION.

What would a nurse in England say to a ward in St. Stephen's Hospital at Delhi, whose temperature the other day was 108 deg. by day and 102 deg. by night, the air being full of hot dust at the same time? The native nurses in this hospital are all Christians. The dispensary is usually well attended, the in-patients being chiefly drawn from this department. A class is held in the hospital for the training of native midwives—a most useful and necessary labour, and not an easy one, for the work is looked upon as "dirty" by the native mind, and sick people generally are regarded as unclean. On the Queen's birthday all the nurses and children and some of the convalescents had an outing in some beautiful gardens outside Delhi. The treat was immensely enjoyed, the fireworks being a particular attraction.

### LEICESTER INFIRMARY CHAPEL.

A beautiful window has just been put up in this chapel. It is in memory of the late chaplain, the Rev. S. Godber. The figures in the window are those of St. John and St. Barnabas; the inscription below records the long work of the chaplain, who is described as "a true son of consolation." The lady superintendent of the infirmary wishes to state how greatly she appreciates the response made by Leicester nurses, past and present, to her appeal for help towards funds for the window.

### SLANDERING A NURSING HOME AT MANCHESTER.

A remarkable vindication of the character of a nursing home was recently made at the Manchester Assize Courts. The proprietress and manageress, Miss Kate Stewart, of Nelson Home, Manchester, brought an action against Mrs. De Bolivar, whose daughter entered the home for the purpose of undergoing an operation for the jaw. Mrs. De Bolivar, when her child was in the home, was told that she could only see her with permission of the surgeon, Mr. Thomas Jones, and to him she made various statements reflecting on Miss Stewart and her management of the institution. Naturally, Miss Stewart resented these accusations, and requested Mrs. De Bolivar to withdraw and apologise for them. As she refused, the case was brought into Court, among the witnesses for Miss Stewart being Judge Parry, who was one of her patients last year. The Judge gave the most favourable evidence of his treatment, and of the home; and Mr. Justice Bigham, in reviewing the case, declared "that there was not a ghost of evidence that the plaintiff's home was not a well-managed and well-ordered institution." The jury found at once for Miss Stewart, and assessed the damages at £50, judgment being entered accordingly. It should be added that Miss Stewart has been a nurse for over twenty years.

### MORE HUMOURS OF DISTRICT NURSING.

For some weeks the people in a district in the north of England called nurse "'t new wuman," having not the least idea of the present day meaning of the term "new woman." An old woman who was looking after a case of confinement was greatly distressed because the nurse wished to change the patient's clothes, as she thought, too soon, and after a little persuasion on the nurse's part the old woman gave way, saying, in a sorrowful voice, "Well,never mind,you're only new, but you'll soon get into our ways." She was much surprised when the nurse replied, "Oh, no, I have come to teach you my ways." The old lady looked at her, and exclaimed: "Eh, what, a bit of a young thing like you, and me who's bin nursing this forty year, never!" Most of the people are mystified about their nurse's social position, they are constantly asking her why she does not do her own washing and work in the mill; at the same time they cannot make out why she has not their brogue, and how it is she can read and write so well, and visit with what they call "the better end," and most of them have come to the conclusion that "nurses get pushed up in the world."

### SHORT ITEMS.

Last week the Princess of Wales opened the new buildings for the Alexandra Hospital for Children with Hip Disease, Queen Square, Bloomsbury. The hospital started, 32 years ago, with ten beds; it now contains 60. The Prince of Wales expressed his satisfaction that the buildings, which have been erected at a cost of £20,000, were opened free of debt.—In confirmation of the statement that the Prince of Wales was well satisfied with his surprise visit to the Princess Alice Memorial Hospital, at Eastbourne, comes the announcement that His Royal Highness has promised to present a new operating table for the theatre to the hospital.—A garden party and fête in the grounds of Holme House, Lightcliffe, on behalf of the Lightcliffe District Nurse Fund, resulted in the latter benefitting to the extent of nearly £40.—Under the will of Mr. Thomas Brandreth, of Devon House, Wimbledon Park, Guy's Hospital receives a bequest of £1,000; the Seamen's Hospital at Greenwich, St. Mary's Hospital, the Middlesex Hospital, the East London Hospital for Children, and the London Fever Hospital, £500 each; the Royal Free Hospital, £250; and the Stanley Hospital, Bootle, the Liverpool Royal Infirmary, and the Liverpool Southern Hospital, £100 each.

### Gynæcological Nursing.

By G. A. Hawkins-Ambler, F.R.C.S., Surgeon to the Samaritan Free Hospital for Women; Assistant Surgeon to the Stanley Hospital, Liverpool.

(*Continued from page* 220.)

### THE USE AND CARE OF THE CATHETER.

For general use, the glass catheter is the most satisfactory. You can easily see that it is clean, and, being made of glass, it can be not only washed and boiled and so sterilised, but may be soaked in antiseptics as strong as commercial nitric acid without damaging it. The latter, however, is not a desirable thing to use, because it is difficult to handle without damaging clothing or fingers. But a catheter made of toughened glass is as reliable an instrument as can be obtained. Sometimes silver catheters are used, sometimes we use gum elastic, and the ordinary male gum elastic catheter, about No. 8 in size, is frequently more easy to manage than a glass one. It is an instrument with which the greatest care is necessary to ensure its being absolutely clean in the surgical sense, because an unclean catheter introduced into a healthy bladder would, in all probability, excite troublesome inflammation, a complication of a very trying nature in any case. Like the other internal surfaces of the body, that of the bladder is very prone to absorb poisonous matter, and to suffer from the presence of infective germs. You will therefore never use an instrument that has not been sterilised, either by boiling or by soaking in carbolic acid, 1 in 20; corrosive sublimate, 1 in 500 to 1,000; or iodine water of the colour of sherry. Of all these, boiling is the best. After use, the instrument must be again thoroughly washed under a stream of running water and replaced in antiseptic solution, or boiled before use. Sometimes it is advisable to attach to the end of the catheter a convenient length of rubber tubing, which must, of course, be as thoroughly disinfected as the instrument itself. By this means you are able to convey the fluid into a convenient receptacle with less disturbance to the patient. Before using it you must not only be satisfied as to the cleanliness of your instrument, but you must take care that your hands have been thoroughly scrubbed and soaked in some disinfectant solution, and where the patient is suffering from any purulent discharge, or where the parts are covered with blood or secretion, it is necessary to wipe this away with absorbent wool soaked in disinfectant before passing the catheter.

The patient will be best lying on her back close to the right side of the bed, with her knees drawn up. You will stand on her right side, and have, either between the legs or on one side of her if your catheter tube be long enough, a warm basin or soap-dish to receive the urine drawn. You will then lubricate the finger of your left hand and pass it over the woman's right thigh to reach the vulva. Just above the opening of the vagina you will feel a small dimple. This dimple is caused by the opening of the urethra, which dips in here and is surrounded by a firmer fibrous ring. Your catheter, already warmed and lubricated with carbolic oil, 1 in 15, will now be passed under thigh with the right hand and pressed gently against this dimple in a direction upwards and backwards, and it will readily slip along the urethra into the bladder, a distance of about one and a half inches. When all the urine has been drawn off you will pinch the end of the tube or place your finger over the end of the catheter, withdraw it gently, and let the urine remaining in the tube run into the vessel you have ready.

In some cases it is difficult to find the opening of the urethra, and it will be necessary to separate the labia and look for it. This is a good thing to do also after operations in this region, where you are liable to have blood and discharge of various sorts about, which it is desirable should not be pushed into the bladder along the point of your instrument—a thing easily done if there is any fumbling with the performance of this simple operation. If your instrument passes with unusual facility and there is no appearance of urine, you have probably slipped it into the vagina. In such cases, and where you do not readily hit the opening of the urethra, you must clean your instrument again by dipping it into solution.

Another way of passing the catheter is by introducing your finger just inside the vagina and pressing it gently upwards. You have now between your finger and the bone the passage into the bladder, and if you run along your finger the catheter, it will in most cases almost of necessity strike the opening of the urethra and be easily introduced.

At times it is necessary to wash out the bladder, and this is easily done by applying a glass funnel to the end of a piece of rubber, which is attached at its other extremity to the catheter. Having filled this with the injection, you will pass the catheter into the bladder and gently raise the funnel for a few inches, by which means the fluid will pass into the bladder. Sometimes you will have provided for you a double-way catheter, so that as you pour the fluid one way it passes into the bladder, and runs out through the other opening into the vessel prepared for it. Where you have not one of these useful instruments, you will, after pouring from one-half to one pint of the injection into the bladder, capsize the funnel below the level of the patient and let the injection run back through it into the vessel.

Boric acid, from two to four drachms in a pint, is a common injection for this purpose, and the water to be used will be of a temperature of 100 deg. F. Of course, the surgeon in attendance will instruct you as to the quantity, the strength of the injection, and the frequency with which you are to use it.

Where a gum-elastic catheter is used, it must also be most carefully washed after use, and kept soaking in carbolic lotion until required again; in my own practice I do not permit such an instrument to be used more than six times without changing it for a new one.

You will observe the character of the urine that is drawn off, and, in washing out the bladder, the nature of any deposit that comes with it; and in case of any peculiarity about the urine you will save it in a clean, covered glass vessel for inspection by the surgeon, whether you have instructions to that effect or not.

Occasionally we have to obtain urine by the catheter, not because the patient is unable to pass it, but to avoid its mixture with discharge from other sources, which would obscure the examination of the urine, and it is very necessary to keep the specimens in clean vessels and secure their examination at the earliest possible moment.

### Appointments.

Lytham Cottage Hospital.—On July 19th Miss M. A. Seller was appointed Matron. She was trained at Oldham Royal Infirmary, and has subsequently held appointments at Manchester Royal Eye Hospital and the Seamen's Hospital, Greenwich.

Bromley and Beckenham Joint Hospital.—On July 14th Miss S. I. Glanville was appointed Matron. She was trained at St. Bartholomew's Hospital, London, and was assistant matron of Hackney Infirmary, Homerton, N.E.

### A Year's Plague Nursing in India.

By a Sister.

### NURSING EUROPEANS AT BOMBAY AND NATIVES AT KARRACHI.

It has probably struck my readers that there may have been some difficulty in disposing of the vast number of people who died in the hospitals. The bodies of the natives had to be dealt with according to their religion. For instance, the Hindoos were burnt and the Mahommedans were buried, and it is a pity that cremation was not made the rule without an exception as it was by far the best and safest mode. Our camp was close by the river, on the banks of which the bodies of the Hindoos were burnt in the way represented in the picture, but the funeral pile was also placed over the body, covering it entirely, and in six hours the remains were reduced to ashes.

When plague was raging and the death rate was very high, sometimes twenty of these funeral piles were burning at a time, the bright flames of which could easily be seen from our camp. These burnings went on all night, and the next morning there was nothing left but a few ashes. The burial ground was also just outside the camp, and each body was placed six feet into the ground and buried with quicklime. It was, indeed, a heartrending sight to see these poor nameless graves so near together, one vast sea of death and ruin, and it made one realise to the full that "in the midst of li e we were in death."

After spending a short time at the hotel to enjoy a little rest and holiday, four of us received orders to proceed to the European plague camp which had very quickly been rebuilt. Here I was placed in charge, and I must confess our work there was very hard. We began by taking in a few convalescent plague cases, but very soon others began to come in and we became very busy. To nurse Europeans required a great deal more time and trouble than nursing the natives, as, of course, we could not let the latter do as much for our English patients as they could so well do for each other. Consequently our work was infinitely more trying. We also greatly felt the heat in Bombay. The sun all day was burning hot, and there was not much shelter in the camp. The plague amongst the Europeans only lasted a short time, and counting all the cases I do not think we had more than twenty-eight or thirty, of which we must have lost eight. Most of the Europeans had a fairly mild form of the disease, but those who died of it suffered as acutely as any native plague case I have ever seen. The majority of the fatal ones were either of the pneumonic form or had acute cerebral trouble, and in one instance a small child of three had the parotid glands involved. Plague is said to be carried from house to house by rats, and an instance of this may be seen in the following case. Strange to say that seven of our European plague cases came from one house, three of them proving fatal, and, on the place being thoroughly examined, dead rats were discovered to be in the flooring, and there is no doubt this must have been the cause of it. Owing to the recent fire all the hospital linen and property had been burnt, and as at that time there were very few cases it was not thought necessary to go to the expense of getting more than was absolutely needed, so that when we took charge of the camp and fresh cases began to arrive we had by no means a good stock of things, and had to get them with great difficulty as quickly as possible. Just as everything was again in fairly good working order no further cases appeared, so the camp was left in charge of two nurses, and we received urgent orders to proceed to Karrachi, where, if anything, plague was raging worse than at Bombay. Considering the size of the place the epidemic was truly awful, and here again we had to deal with a very strange set of people, whose manners and language were still more hard to understand. The Karrachi Hospital, which was built of stone, was given over for plague cases; the centre part was double-storied, and the nurses occupied the rooms upstairs. In a very short time it was plainly seen that it was not nearly large enough to hold the number of patients who came pouring in. Accordingly, all round the grounds, sheds of matting and bamboo were quickly erected, and very soon every ward and every shed was full. The plague authorities arranged that no nurses should be kept on duty night or day for longer than eight hours, which was very thoughtful of them. Even then it was very trying work, and we found that, owing to the noise, heat, and light, it was no easy task for those on night duty to be able to get sufficient sleep in the daytime. In Karrachi we again experienced the usual difficulty of overcoming the prejudices of the people, and we had the same discouraging treatment when we were so anxious to do our very best for them. Indeed, I have often said, and firmly believe, that some of our patients died of "high caste." This sounds strange, but so much that we actually had to do for them was strictly against their religion, and in some cases I myself have seen natives drawing away and hiding their food in case of our

Burning the Hindoo Patients after Death.

shadow falling across it? It was not until after some little time, when they saw other patients in the wards being nursed and looked after, that they began to believe in us. Later on, in my own experience of nursing, I found that we managed in the end to win them over completely, and not merely did they allow us to do what was necessary for them, but they liked and respected us. Indeed, they would often take their nourishment from the nurses when they refused it from their own people, and were always grateful for any trouble we took in respect of them. Instead of being received with looks of dislike and distrust they began to look forward to seeing us, and it was quite a pleasure to visit our wards and to be met with looks of absolute trust, affection, and friendliness. It was worth all the trouble and sacrifice of which we were capable to see the true delight on the faces of those in the convalescent wards. Poor souls! they had, many of them, passed through terrible suffering, and had been very near "the gate of death." I have often been asked if the natives appreciated our nursing them, and if they were grateful for all that was done. Well, according to my previous account as to the way they behaved, it appears they were anything but nice; but, after all, there was every excuse for them at first, and if they rebelled it was only on account of their religion, which, perhaps, may be a mistaken one, but nevertheless they are more true to it than many a so-called Christian. In order to make the natives happier and to let them see the treatment of the cases in the hospitals, it was arranged that each patient might have one relative at hand who could come and see them whenever they liked, and sheds were built close by for the accommodation of these people. In many instances they stayed with their relatives until the end, Government providing them with free quarters and food. This had to be done to quell the fears of the people, and although there was a grave risk of thus spreading the disease, yet at the same time it was hard to completely isolate the patients when there was every chance of their not recovering. Moreover, it was a great comfort for the natives to feel that they could see their own sick folk and watch beside them if they so wished, and I think it was a very wise as well as a very kind arrangement. The natives do not appear to fear death in the least, and I have been often struck at their marvellous composure. Their patience and endurance in sickness is also truly wonderful, and it was pathetic often to see them pretending to feel better and to say they were "*atcha*," which means "well," just to please us. Plague nursing was one of the saddest experiences I have ever known, as it was a daily lesson to us of suffering patiently and bravely borne. Yet, in spite of all its drawbacks, disappointments, and sadness, it was one of the years of my life I shall always look back upon with true pleasure.

### TRAVEL NOTES AND QUERIES.

Switzerland for a Month (The Matron).—I fear you have set yourself an impossible task. It is out of the question to go to Switzerland for a month, much less six weeks, on £10, especially in the height of the season. To begin with, the journey swallows more than half the amount. You could live at Meiringen for 6 francs a day, which is 5s., making 35s. a week. You cannot possibly reckon on less than 5s. extra for tips, washing, and other small expenses; your journey would be, second class return, £5 9s. 6d.; this only leaves you £4 10s. 6d. for your month's stay. I fear it cannot be done, unless you can stretch your resources a bit. There are many places that will take you for 5 francs in Switzerland, but not for the month of August. If you think you can manage this, write me again, and I will tell you of several places to make a choice. Remember health should come before everything.

Paris in September (Alys).—It is not easy to find any place in Paris where they will take you only for bed and breakfast, and most certainly not an English-speaking place. The only place I could recommend is the little Hotel Britannique. I do not remember the name of the street, but it is well known. There you might arrange for bed and breakfast. Then there is Madame Mansfield, 157, Faubourg St. Honoré, whose inclusive terms are from 5 or 6 francs; or Miss Willoughby, 4, Rue des Pyramids, about the same terms. The two latter English.

### Lichfield Victoria Nursing Home.

This institution, which is the local outcome of the two Victorian Jubilees, so far as the City of Lichfield is concerned, was formally opened on Monday by one of its chief benefactors, Mr. S. Lipscomb Leckham. For years past a nursing organisation has existed in Lichfield, but its location has been temporary, and, therefore, often inconvenient. The late Archdeacon Scott had in his lifetime given attention to the matter, and, outside the Jubilee festivities, the subscriptions then initiated have been for the provision of a permanent home. The questions of both site and building have been earnestly, and at times warmly, considered, but a majority of the committee decided on acquiring a substantial house in the centre of Lichfield, which they have purchased, fitted, and furnished in a complete manner. The objects in view are: The free nursing of the sick in their own homes; an invalids' kitchen; while two wards are to be used for emergency cases of accident, &c. The freehold has cost £1,003, and nearly £200 has been spent in furnishing, &c. At the opening ceremony there was an influential and representative gathering. The Dean of Lichfield, the Rev. Canon Lonsdale, the Sheriff of Lichfield, Mr. A. O. Worthington, Mr. R. P. Cooper, the Rev. Prebendary Bolton, and others took part in the ceremony. The executive committee who had undertaken the furnishing were warmly congratulated on the success of their efforts. They had the assistance of Miss Graham, Miss Scott, and Nurse Harding, who is now practically the matron of the establishment. The neighbourhood was brightly decorated for the occasion, and the Lichfield Jubilee Memorial had a most successful and promising "send off."

### Minor Appointments.

Worcester General Infirmary.—Miss Ada Pratt has been appointed Sister of the children's ward. She was trained at the Victoria Hospital for Children, London, and at the York County Hospital, and has since done private nursing for three years on the staff of the Victoria Hospital Private Nursing Home.

Her Majesty's Hospital, Stepney.—On July 24th Miss Octavia E. Rose was appointed Charge Nurse. She was trained at the Blackburn and East Lancashire Infirmary, and has since held the posts of sister of the male medical ward, Blackburn Infirmary, and charge nurse at Ilford Isolation Hospital.

Burton-on-Trent Infirmary.—On July 12th Miss Theodora Nicholson was appointed Charge Nurse of the Children's New Ward. She had three years' training at the Oldham Infirmary, and has since been private nurse at the Oldham Nursing Association for a year and nine months.

Monsall Fever Hospital, Manchester.—On July 20th Miss Maud Goldsmith was appointed Sister. She was trained at St. Thomas's Hospital for four years—first as probationer, and since as staff-nurse.

Tonbridge Union, Kent.—Miss Alice Owens has been appointed Assistant Nurse. She was trained at St. Peter's Home, Mortimer Road, Kilburn, by the Meath Workhouse Attendants' Association.

Western Fever Hospital, Fulham.—On July 25th Miss Florence Farrar was appointed Charge Nurse. She was trained at the Huddersfield Infirmary, and subsequently for ten years was charge nurse at Ramsgate General Hospital.

Holborn Union Workhouse.—Miss Jessie Oake has been appointed Head Night and Midwifery Nurse. She was trained at Greenwich Infirmary.

### Royal National Pension Fund for Nurses.

### PRESENTATION OF CERTIFICATES.

It is just four years ago since last the Princess of Wales, President of the Royal National Pension Fund, presented certificates to her nurses. On Friday, July 26th, 1895, between three and four thousand nurses had joined the Pension Fund since its commencement. On Friday, July 21st, 1899, as many as 7,000 nurses had joined its ranks. This does not mean that there are actually now 7,000 members in the Pension Fund, for marriage, good fortune, misfortune, or, alas! death, has caused some members to fall out of the ranks, and the number a nurse vacates is not bequeathed to a successor.

When Her Royal Highness most graciously expressed her desire, on the present occasion, to hold a fourth presentation as many as 2,389 nurses were eligible to receive their certificates, and 2,347 invitations were actually sent out. It is a remarkable fact, considering the nurses were engaged in pursuing their calling in all parts of Great Britain and Europe, that upwards of 1,000 were able to avail themselves of the much-prized privilege of receiving their certificates at the hands of the Princess. In many cases the nurses had to exercise much self-sacrifice in order to be present. One nurse travelled overnight from Paris, returning again as soon as the ceremony was over. Private nurses especially found it difficult to leave their patients, and hurried from long distances as speedily as possible without regard to fatigue, fully satisfied with the compensating pleasure of a day at Marlborough House and a sight of "Our Princess."

### In the Garden at Marlborough House before the Ceremony.

The hour at which the nurses were invited to assemble in the gardens of Marlborough House was half-past ten. Long before that hour all the approaches to the entrance-gates were thronged with hundreds of nurses, and an interested and wondering crowd assembled to watch the arrivals. Indoor uniform was worn where possible, whilst the Princess's armlet adorned the left arm. A large cloak-room had been provided in the grounds, and this was soon thronged with nurses divesting themselves of cloaks, bonnets, umbrellas, baskets, bags, and what not. Then an army of trim nurses, in every variety of uniform, dispersed under the cool shade of the trees, or visited the refreshment tables, temptingly spread under shady awning. Soon twelve sergeants, under the command of General Crease, C.B., arrived to conduct a rehearsal of the ceremony to take place at half-past twelve.

### The Drill.

Large cards raised on polls marked the position of each company of a hundred nurses, and indicated the numbers which corresponded with those marked on the card held by each nurse. A sergeant was placed in charge of each company, and so well did the nurses respond to the directions given them that within a quarter of an hour all were in their places and ready to move in correct order about the grounds under the guidance of the sergeants. The experimental march of each company of nurses was an exceedingly pretty sight. Four abreast, and arm in arm to keep the line, the nurses, in white caps and aprons, formed a very charming picture wending their way from the cool shade of the trees into the bright sunshine and round the terraced walks back into shelter again, to form behind their standard once more. Each company thus in its turn was prepared for what was required of them later on. When the result of the rehearsal was pronounced satisfactory formality was somewhat relaxed. The sergeants, in the most thoughtful and polite manner, provided the nurses of their respective companies with chairs, or conducted them to the refreshment tables, for the day was very hot, and many had been unable to procure proper refreshment before an early start.

### The Badges and Uniforms.

Whilst the nurses were thus assembled and awaiting the appointed hour for the ceremony the best opportunity was afforded for observing uniforms, badges, and medals, and their interesting owners. Blue was largely represented amongst the uniforms, but otherwise it was impossible to say that any one colour predominated. There were stripes of all sorts, a certain number of stuff dresses, and most of the caps and aprons were neat and becoming. The two army sisters, with their delightful little scarlet capes and folded handkerchief head dress, of course came in for a large share of notice. The Berrywood Asylum nurses, whose uniform is still more military in effect than that of the army sisters, naturally were not overlooked. Many and varied were the medals and badges displayed. Some nurses carried as many as four such adornments. Several were actual rewards of merit, whilst others simply denoted that the wearer was connected with some public or private institution. We noticed the gold medal of St. Bartholomew's Hospital, worn by Miss Turner, the first nurse to possess this much-coveted honour; also the Maidstone typhoid epidemic medal of silver, presented to the nurses who so valiantly battled with the disease during the terrible outbreak, worn with a blue and yellow ribbon. The Army Nursing Reserve badge, a large round silver medal with a cross in the centre, looked very handsome. The medallions of the Guild of St. Barnabas and the Queen's Jubilee Institution worn suspended from the neck were very effective; and the badges of the Royal British Nurses' Association (very largely represented), the Westminster Hospital, Workhouse Infirmary nurses, and North Staffordshire Nurses' Institute were all much admired.

### Awaiting the Ceremony.

Soon after twelve o'clock the invited guests began to arrive. They were mostly presidents and lady presidents of the League of Mercy, the latest institution formed by the Prince of Wales to assist the hospitals, and members of the Council of the Pension Fund. Amongst those present were:

Sir M. M. Bhownaggree, M.P., Mr. J. Allen Baker, Mrs. Boulnois, Sir Henry Burdett, K.C.B., Lady Burdett, Miss Burdett, Mrs. Boscawen, Mrs. Walter H. Burns, Sir William and Lady Broadbent, Mr. F. S. W. Cornwallis, M.P., Mr. Walter S. M. Burns, Sir W. H. and Lady Crundall, Dr. and Mrs. W. J. Collins, Miss Mabel Cave, Mr. Goddard Clarke, Mr. and Mrs. J. G. Craggs, Major-General Crease, C.B., Mrs. Moss Cockle, Mr. and Mrs. L. H. M. Dick, Miss Mary L. E. Dunn, Mrs. Dibdin, Lady Emily Hart Dyke, Major-General and Lady Evelyn Ewart, Lady Ebury, Lady Ellis, Mr. E. Baxter Forman, Mrs. Bretland Farmer, Miss E. Fisher, Miss Forman, Lady Farquhar, Mrs. Farrar, Mrs. Farmer, Colonel Ford, Mr. Ralph Gooding, Mr. F. Green, Mr. E. Gray, M.P., Mr. Frederick Gordon, Mr. Tyrrell Giles, M.P., Mrs. Tyrrell Giles, Miss L. M. Gordon, Mrs. Gooding, Mr. C. T. Harris, Mr. W. Haydon, Mr. and Mrs. Gresley Hall, Mr. John Harris, Colonel Hughes, M.P., Mr. G. B. Hudson, M.P., Mr. J. and Miss Hutchinson, Mr. and Mrs. C. Eric Hambro, Mr. and Mrs. L. V. Harcourt, Mr. J. Harrison, Mrs. Edwin Hughes, Miss Harrington, Mrs. Harrison, Mr. W. H. Keay, Mr. and Mrs. George King, Colonel Knollys, Right Hon. the Lord Mayor and Lady Mayoress of London, Sir Fitzroy Maclean, Bart., C.B., and Lady Maclean, Sir Jos. Sebag-Montefiore, Miss Tessa Mackenzie, Miss K. M. Monk, Mr. and Mrs. J. Pierpont Morgan, jun., Mrs. McIntosh, Mr. and Mrs. G. Norman, Colonel Arthur A. Owen, Mr. J. B. Porter, Mr. Ambrose Pomeroy, Sir W. D. Pearson, M.P., Mr. G. W. Potter, M.D., Miss R. Pritchard, Mr. J. Round, M.P., Mr. and Mrs. Edward Rawlings, Mr. Alfred Charles de Rothschild, Mrs. Gurdon Rebow, Mr. Montague Sharpe, Mr. H. C. Stephens, M.P., Mrs. Sandars, Mrs. Stephens, Mr. B. S. Strauss, Alice, Countess of Strafford, Alderman W. P. Treloar and Mrs. Treloar, Mr. T. McKinnon Wood, Mr. W. B. Whittingham, Mrs. Wightman, Miss Wedgwood, Mr. W. B. Yates, Marchioness of Zetland.

Whilst the guests were assembling, and during the afternoon, the band of the Royal Horse Guards played in the grounds.

### The Ceremony.

Punctually at half-past twelve the Princess descended the steps from Marlborough House leading into the garden, and took her place under the scarlet canopy supported on silver poles, which had been erected on the lawn for the purpose. She was accompanied by the Prince of Wales, the Duchess of York, and Princess Victoria of Wales, and by ladies and gentlemen of the household. Little Prince Edward of York, in sailor attire, joined the party later. As soon as the Princess, who was charmingly dressed in black, had taken up her position, she proceeded to present certificates of membership to the following matrons: Miss Smedley, St. George's Hospital; Mrs. Price, Hospital for Consumption, Brompton; Miss Esther Young, assistant matron Guy's Hospital; Miss E. Wilkinson, Derby Royal Infirmary; Miss Easton, Royal Berkshire Hospital; Miss Morris, Bristol General Hospital; Miss Herbert, General Hospital, Worcester; Miss Chambers, Ancoats Hospital, Manchester; and Miss Smith, Guy's Nursing Institution.

Then came the nurses. Sir Dighton Probyn introduced each nurse by name, and Sir Henry Burdett handed the certificates to the Princess. The nurses passed in single file, each bowed to the Princess, who, in presenting the certificates, never failed to smile sweetly at every nurse. Although the ceremony could not fail to be attended with much fatigue, for the nurses numbered upwards of 1,000, the Princess showed a kind and unflagging interest throughout, and when, owing to a mistake, there was some delay in finding the certificate of one nurse, Her Royal Highness exhibited not the least impatience on her own account, but expressed her concern that the "poor nurse should be kept so long waiting." Acceptable as the pity was, the object by no means needed it. She was the envied of her fellow-nurses. All had been obliged to pass the Princess as quickly as possible. Here was some one favoured by fortune to stand near "Our Princess" for some time, and be the object of her special attention!

All had been so well arranged by the officers of the Fund, those who had charge of the arrangements at Marlborough House, and the military assistants that with this one incident the presentation was carried through like clockwork in the remarkably short space of 35 minutes!

When the last certificate had been given, the nurses, who had formed a semi-circle in front of the pavilion, at a motion from the Prince of Wales drew nearer that they might hear the speeches which followed.

### The Speeches.

It had been announced that Mr. Hambro, chairman of the Pension Fund, would offer the thanks of the nurses to the Prince and Princess of Wales. Illness, however, prevented Mr. Hambro from attending, and Sir Henry Burdett, deputy-chairman and founder, addressed their Royal Highnesses in the following words:

In the regrettable absence through illness of Mr. Hambro, chairman of the council, the pleasant duty has devolved upon me of tendering to your Royal Highnesses on behalf of the council, officials, and nurses their most grateful thanks for the generous way in which you have received the nurses, and to the Princess, who has so graciously presented them with their certificates and armlets, which make them the Princess of Wales's own nurses. I have further to thank your Royal Highnesses for your loyalty to the Fund from the outset, whereby all difficulties and opposition have been removed, and complete success attained. It is not too much to say that the cordiality with which Her Royal Highness the Princess has identified herself with the Pension Fund from the outset has made it the most influential and successful of insurance and friendly societies for women which the world has ever seen. The Fund commenced in 1888 with the sum of £20,000 and 500 nurses. When Her Royal Highness presented the certificates to the third and fourth thousand nurses four years ago the invested funds amounted to £200,000 and nearly 3,000 policies had been issued. To-day the invested funds amount to nearly half a million of money, and 7,250 policies have been issued. Nor is this all, for during the four years which have elapsed since Her Royal Highness was graciously pleased to receive the third and fourth thousand nurses, no less than £250,000 has been paid into the Fund by the nurses themselves. The income of the Fund at the present time is £83,000 a year, and during the last four years the Council have distributed amongst the nurses some £6,000 in sick pay, and nearly £3,000 in addition has been given to nurses who found themselves in temporary difficulties under the direction of Lady Rothschild, the president of the Benevolent Fund Committee. There has been nothing like it before in the history of insurance and friendly societies. Nurses working in every part of the British Empire are eligible for membership of the Fund. Among those who were entitled to come here to-day, but who are prevented by the remoteness of their stations, are nurses in service in Australasia, South Africa, Egypt, Mauritius, India, China, Fiji, the West Indies, and in the United States. This will show the all-embracing nature of the benefits and protection which the Fund affords to British nurses everywhere. Your Royal Highnesses give so much of your time to the cause of benevolence and the promotion of the best interests of worthy charities of all descriptions that you may consider me too bold when I state my earnest conviction that among all the enterprises to which you have extended your support, I do not believe there is one more beneficial in its influence or more helpful to a large number of devoted workers amongst the sick and suffering, and there is certainly not one which in so short a time has proved so successful as the Royal National Pension Fund for Nurses, of which the Princess of Wales is president. Looking back to the past history of the Fund it is manifest to all connected with the management, and especially to the members of council and the officials, that the support extended to the Fund by your Royal Highnesses from the outset is undoubtedly the chief cause of the splendid success which has been achieved. I can assure you of our gratitude and sense of indebtedness. I am confident, too, that when the nurses return, as they will shortly do, to their duties of ministering to the sick and suffering in our hospitals and private houses, the pleasant remembrance of this gracious reception at Marlborough House to-day will be ever with them, to cheer them in their work, to encourage them to be better women, and if possible to spur them on to be even better nurses too.

Sir Henry's speech was received with applause, and at its conclusion he asked the nurses to express their own feelings by cheers first for the Princess and then for the Prince of Wales. Very hearty was the response that immediately followed, and Hip, hip, hurrah! rose most enthusiastically, if not very powerfully, from the assembled throng. When the last sound had died away the Prince responded to Sir Henry Burdett's address.

Sir Henry Burdett,—The Princess of Wales desires me to thank you very much for the kind words you have just spoken and the vote of thanks you have just accorded her for distributing on the fourth occasion the certificates for these nurses. You have given us most interesting and valuable statistics, which relieve me of the necessity of saying more on that head. But I think I may say this, that I quite agree with you that there is no philanthropic object with which we are more gladly associated than this Pension Fund for Nurses. I rejoice to see that the members are increasing so enormously, and from the sum of money you have now got there is little chance but that the Fund will always remain on a solid basis. To you especially, Sir Henry—I am glad to have the opportunity of saying it—are due the thanks of all for your having done so much in starting this scheme, which has now become one which will, I am sure, exist in perpetuity. The Princess of Wales and myself are very glad to have welcomed you all here to-day in our garden. We trust you will be none the worse for your long journey here and back in this very hot weather, and we now hope you will like to walk about and amuse yourselves and get a little refreshment.

### After the Ceremony.

Many of the nurses responded at once to the kind and thoughtful invitation of the Prince to make use of the charming garden; but others lingered to watch the reception of the assembled guests which took place immediately after the presentation of certificates. First came the presidents and lady presidents of the League of Mercy, in which the Prince takes so keen and personal an interest, and then followed those connected with the Pension Fund. When all had been welcomed by the Prince and Princess, the Royal party retired. Many nurses remained long afterwards resting in the shade and enjoying the pleasant refreshments provided. It was interesting to see how desirous the nurses were to take away some memento of the delightful occasion. The tables were decorated with roses at the commencement of the proceedings, but not one remained to shed its perfume unnoticed by the time the nurses dispersed to their various destinations. Instead, they rendered a festive appearance to white aprons, or were carefully carried in the hands of the nurses to be treasured as a souvenir of a never-to-be-forgotten red-letter day in the lives of the possessors. Thus ended one of the most interesting events of the present season—one that will bear good fruit for many a long time to come; for in it were united the tender sympathy and interest of the Royal lady for those whose mission it is to help the sick and suffering, and the loyal devotion and loving gratitude of those who not only are proud to call themselves "her nurses," but who, as members of the Royal National Pension Fund, represent a splendid outcome of the present century—women who are self-denying, self-respecting, and prudent.

---

### SOME OBSERVATIONS BY AN ONLOOKER.

The unique ceremony which took place at Marlborough House on Friday was remarkable in many ways. As Sir Henry Burdett remarked in his address, the Royal National Pension Fund for Nurses represents the most successful co-operation amongst women the world has ever seen. Its establishment marks an epoch in the history of womankind. When Sir Henry Burdett, in 1886, first conceived the idea of such an organisation, opinion as to the capacity of women to unite and maintain any self-supporting provident institution for their benefit was evidently at its lowest ebb. The proposal was laughed to scorn by the majority. Fortunately there were those who believed that, generously supported and encouraged, women as well as men would gladly recognise the immense advantage of independence and comfort at the end of a working life. Those who, in the face of scepticism and opposition, stood by the nurses of England in 1886, and by their disinterested munificence enabled the Pension Fund to be established, might well be proud of the result presented on Friday last. The ceremony at Marlborough House testified to a success beyond the highest anticipations. It witnessed to a vast body of the women of England representing one calling only, earning an average living wage of but moderate amount during a very limited working life, who by their prudence and self-denial had rendered themselves independent of their friends or the assistance of the State. It showed, moreover, the excellent effect of well-regulated and disciplined employment upon the feminine character. The excellent manner in which the thousand nurses united as one in cheerfully and intelligently co-operating with those who so kindly undertook the very novel and somewhat puzzling task of drilling female recruits to be ready for service in one day provoked both surprise and admiration.

General Crease, C.B., has, from the inauguration of the delightful ceremonies at Marlborough House, been in charge of these female battalions. Some decade has elapsed since the first of these took place, and advance is reported all along the female line. In commenting upon Friday's arrangements, General Crease remarks: "The thing that strikes me most is the fact that in less than 15 minutes after we commenced upwards of 1,000 women, strange to each other, to the sergeants and to me, should have been so disciplined as to be capable of being moved anywhere on the grounds without noise or confusion, and that these women, many of them quite undisciplined and unaccustomed to it, being private nurses, should have been brought under absolute control in so short a time. I am surprised, and so I think were the sergeants, who did their work admirably." Coming from such an authority as General Crease, this opinion is, indeed, encouraging, and it is one that must have been endorsed by any observant spectator. In addition to the words we have quoted above, General Crease strikes another key-note. We think it will serve to emphasise the accuracy of his observation. He says: "I think that these nurses must have a very great respect for the Princess of Wales, as well as a love for her. I can't account even now for their amenability to discipline save as resulting from an intense desire to show their appreciation of Her Royal Highness's kindness, and this was the only way they could prove it. The result was really remarkable." How truly General Crease has interpreted the feelings of the many loyal hearts assembled on Friday only they themselves can tell! Those who went among the nurses that day could gather in some small measure the love and veneration of all for their president, "Our Princess." Her kindness and thoughtfulness were not lost upon an unappreciative assemblage. Nurses have all learnt some lesson in the school of endurance, and they knew that it was not without cost to herself that the Princess undertook to receive so large a number in the first instance, and carried it through with unfailing sweetness and graciousness through the heat of a summer noon, standing all the time, and never failing to accord to each a smile and personal recognition. They realised throughout the day the many instances of kindly thought bestowed. No guests, however honoured, could have received more attention. The Princess's directions were that everything that could tend to the comfort of the nurses should be provided, and right well were her wishes carried out by those at Marlborough House. All united to make the nurses at home and happy, and a spirit of kindliness and consideration pervaded the atmosphere. In another column we have recounted the Princess's solicitude for the nurse who was kept waiting for her certificate. Yet another instance of her consideration was shown when later it was explained to her that, through misdirection of her card of invitation by friends, one nurse had arrived too late to receive her certificate. She immediately consented to receive the nurse by herself and hand her the certificate. No wonder the nurses love their President! Every time she comes personally into contact with them they realise more deeply her kindness and consideration. A skilful and tender nurse herself, familiar with sickness and sorrow, her tender heart goes out to those whose high mission it is to sink all thoughts of self in alleviating the sufferings of others.

### Colonial Nursing Association.

### ANNUAL MEETING.

By the kindness of the Duke and Duchess of Sutherland, the annual meeting of the Colonial Nursing Association on Tuesday afternoon was held at Stafford House. Lord Loch, the president of the society, who was in the chair, briefly traced its history. It originated in the energy of Mrs. Piggott, wife of the Governor of the Mauritius, who, seeing the great need of skilful nursing, and the lamentable number of preventable deaths through lack of it, arranged for two trained nurses to begin work in that colony. The success of the experiment was immediate and marked, and a number of friends rallied round her in order to extend the beneficent operations of the scheme to other colonies. The Association, which was formed in 1896, received every support from the Colonial Office, and from the Government officers in the colonies, whilst the Secretary for State had instituted an important extension by organising the London School for Tropical Diseases at Greenwich. Four nurses have been sent there for six months' training; on the completion of that term they will proceed to the West Coast of Africa, or to some other equally unhealthy locality. The Council had made up their minds to obtain and invest £5,000, so that the subscriptions and guaranteed income would assure the continuance of the society's operations. As £2,000 had already been collected, all that now remained to be done was for their friends to raise £3,000 more, and to strive hard to interest others in their work.

Mr. Chamberlain then moved a resolution approving and confirming the annual report of the Colonial Nursing Association, and pledging the meeting to give its earnest support to the Executive Committee in its endeavour to raise the funds of the Association to £5,000. His speech is reported *verbatim* in another part of the paper.

### Mr. Asquith's Testimony.

In seconding the resolution Mr. Asquith said he was glad to have the opportunity of expressing his hearty admiration of an enterprise so admirably conceived, and, as he believed, so wisely conducted as that of the Colonial Nursing Association. It was, he supposed, a truism that great and marvellous as had been the progress in our time in the art of healing, there had been no advance so noteworthy and so beneficent, whether in the actual cure of disease, or what was equally important, the relief of unnecessary suffering, as the development of the practice and the profession of nursing. The function and training of the nurse were now regarded as equally important as those of the physician and the surgeon, and of late years we had witnessed the recognition of that fact, not only in our great hospitals and large centres of population, but in the small towns, and even in our villages throughout the country. This indispensable alleviation of sickness was being brought home to the doors of those whose means did not allow them to supply it for themselves. If we recognised that duty in the case of those at home, how could we be deaf to the appeal of an association which worked on behalf of men and women, fellow-subjects of our own, who, in more remote and less highly-organised corners of the Empire, amid arduous and dangerous surroundings, were developing its resources—men and women, living, as they unfortunately did, under more trying conditions of climate and environment than any we experienced, exposed to immeasurably greater perils to health and to life, and cut off by the necessities of the case from companionship and the affection and solicitude of relatives and friends. The men and women so situated might surely make a great claim on the benevolence of the people of this country.

Sir George Taubman-Goldie, Dr. Patrick Monsen, and Mr. A. L. Jones also supported the resolution, which was carried by acclamation.

Mr. Chamberlain proposed a vote of thanks to the President, who acknowledged the indebtedness of the society to the nurses who so ably uphold its reputation abroad.

### The Nurses at Marlborough House.

By One of Them.

When I heard from the Secretary of the Pension Fund that Our Princess had invited us to Marlborough House to receive our certificates from her own hand, my first thought was, "Suppose it isn't a fine day." Then I remembered that I had heard that this particular function had always been favoured with Queen's weather, and so I hoped for the best. Friday turned out to be a glorious day. The thunder of the previous night had cleared the atmosphere, and even London looked fresh and brilliant.

### Arriving in the Garden.

I found that many had arrived at Marlborough House before half-past nine. We went in at the entrance which leads to the lovely grounds. How grateful I felt for the refreshing sight of green turf and cool shade, for there I knew we must remain until the ceremony at half-past twelve. In the garden we found the kind secretary of the Pension Fund—whom many of us nurses regard quite in the light of a friend—ready to receive us, as well as an imposing array of sergeants and two officers, who conducted the drill in preparation for the ceremony.

### Old Friends.

Between the intervals of drill most of us managed to find old friends whom we had not seen for some time. It was such a pleasant occasion upon which to meet, too. We had all done our best, within the limits which our uniforms permitted us, to array ourselves suitably. Some, indeed, of the nurses, I thought, had gone a little out of their way to "improve" upon their uniform. I saw one who had reproduced her blue cotton gown in cashmere or alpaca, and to this she had added an extra touch of colour by way of a scarlet belt and a paste buckle! Now, I may be old-fashioned, but I think a nurse ought to appear in as workman-like a garb as possible, and represent her institution in the uniform adopted by it. Of course, a private nurse can follow her fancy, and exercise her own taste in any way she pleases.

### The Uniforms.

Taken as a whole, the effect of the numerous different uniforms was very good. The blue worn by the sisters of the London Hospital, which is also used by several other hospitals, was particularly pretty among the green trees. I noticed a very large number of nurses from the London Hospital. They had chartered an omnibus to bring them to the reception. Several private nurses, and those from Queen Charlotte's Hospital, were dressed all in white, and looked delightfully cool and fresh. I heard a matron who had been present on other occasions say that at the last presentation the nurses from Berrywood wore quaint little scarlet caps like a French "Liberty cap," but this time they all had neat close-fitting bonnets.

### A Brave Man.

Amongst all the throng of bright colours and white caps and aprons one figure stood out conspicuously. This nurse wore neither cap nor apron, nor cotton frock, but was adorned by the Princess's armlet. Briefly, he was a man, the only male nurse present, and one of the very few of his sex who have availed themselves of the privileges the Pension Fund offers to nurses of both sexes alike. Very few male nurses in England have the right to call themselves "trained" nurses, for they have not the opportunity of becoming so. Mr. Ernest Godecharle, however, has managed to secure, as an orderly at Netley, and as a nurse at St. George's Hospital, a very adequate training. He is, moreover, a member of the Royal British Nurses' Association, and his enthusiasm for his profession cannot be doubted, since he faced an ordeal on Friday from which most men would most certainly shrink.

### Examples of Self-denial.

I was greatly struck with the proportion of nurses beyond middle age who were present. I suppose I must number myself amongst the younger ones, and as such I could not help feeling surprise and admiration at the amount of self-denial which must be exercised by the older nurses, since the premiums, of course, are very heavy when they can only be paid for a few years before the policy-holders are entitled to their pensions. Even when we join as young as we possibly can it is not easy to pay the premiums every time they are due, if we have entered for a reasonably large pension; and it always means that many little pleasures have to be foregone. But I am digressing. Under such circumstances any little self-denial was likely to be forgotten, for all were thoroughly enjoying the pleasures of the day.

### Arrival of the Princess.

Long before we had become tired of looking about us and watching the various incidents the other guests had arrived, and the band struck up "God Save the Queen," and "Our Princess" stepped, smiling, through the doors of Marlborough House, followed by the Prince, the Duchess of York, Princess Victoria of Wales, with some of the ladies and gentlemen of the household, and the ceremony commenced. Amongst others I noticed the matrons of St. George's Hospital and those of the Derby Royal Infirmary and the Berkshire Royal Infirmary. The other guests seemed much interested in watching the nurses file past, and though most of us were very nervous when the moment arrived to make our bow, we were told we comported ourselves in a most creditable manner. All ceremony was over when once the certificate was in the hands of the nurses, and as our duties were completed we gradually formed a huge semicircle in front of the Royal group and became spectators in our turn.

### The Speeches.

In less than three-quarters of an hour the presentation was finished, and then came the short speeches, which we all thought excellent. Mr. Hambro, our chairman, was to have thanked the Prince and Princess on our behalf, but illness prevented him attending, and Sir Henry Burdett, our deputy-chairman and founder, filled his place. At the end of his interesting little address, Sir Henry surprised our feminine souls by calling for three cheers for the Princess. Well, we hadn't been practised in this, and usually are able to rely on the stronger voices of our male relations to express our feelings of approval. However, the Hip, hip, hurrahs! of the hundreds of nurses came from the heart, and were the genuine outcome of the most loyal sentiments, if not of the strongest voices. Our enthusiasm having thus found an outlet, the Prince replied briefly, giving us a most kind and gracious welcome on behalf of the Princess and himself.

### The End of a Happy Day.

The ceremony being ended, the guests wandered off to partake of the lunch which awaited them, and soon the long tables were thronged, and groups of nurses sat chatting under the trees, thoroughly enjoying the rest and the pleasant scene before them. Many, when they quitted Marlborough House, carried trophies of roses which had adorned the luncheon tables, as mementoes of one of the happiest events in the lives of some, and never to be forgotten by any of the nurses who had the good fortune to be present.

### Echoes from the Outside World.

### AN OPEN LETTER TO A HOSPITAL NURSE.

The story of the little blind boy in the London Hospital who, when the Princess of Wales privately visited the Mellish Ward last Saturday, was led up to her, and addressed her politely, "How are you, Miss?" reminds me of another incident in connection with her Royal Highness's visits to hospitals which happened some years ago. At one of the large institutions devoted entirely to Incurables she was taken to see a woman who, besides being bedridden, was blind and deaf and dumb. A nurse told the patient, by means of the deaf and dumb language manipulated on to the patient's own fingers, that the Princess of Wales had come to see her, and the poor creature understood at once and asked if she might touch the Princess's hand. When the request was translated to the visitor, Her Royal Highness at once took the extended hand, pressed it warmly between her own, and then turning round, with her face quivering and her eyes full of tears, said brokenly, "I can't stop; it is too sad," and it was a few minutes before she was able to recover her self-possession. But the pain—for the feeling of deep sympathy is pain—was, I am sure, willingly borne by the Princess that she might by her presence give pleasure for a few minutes to one whose existence was so near to death in life. Many of you had an opportunity of observing for yourselves the Royal graciousness on Friday, at Marlborough House. I was so delighted you had such a lovely day—even though it was more than a trifle warm—and that everything went off so satisfactorily. I should desperately have liked to have heard what sort of a cheer you succeeded in giving. Women generally have so little practice in "hip, hip, hoorahing," that they do it uncommonly badly, but I do hope on this occasion you acquitted yourselves—well, like men!

---

I wonder if there are many nurses who smoke? I almost fancy not, because tending those who are ill is such an essentially feminine occupation that it must have its due influence on the character, and should create a distaste for anything "mannish" and wanting in refinement. Just imagine, if you can, a patient, burning with fever or sick with pain, ministered to by a woman reeking of stale tobacco smoke; and so strong does the love of smoking frequently become when it is allowed to grow into a habit that those who indulge in it will do anything rather than give it up. I know a few cases in my own experience. In one, a girl who stayed with me in the same house used to retire, ostensibly to read and write in her bed-room every afternoon, and always returned smelling horribly of the weed which is not necessarily fragrant. Upon one occasion I was obliged to go into the room to fetch something. Knocking two or three times and failing to get an answer, I entered, to see the girl stretched on her bed asleep, her hand extended, and between the loosened fingers a half-consumed cigarette, which had apparently been extinguished by coming in contact with the counterpane. After that I was prepared for a fire any afternoon, and was quite surprised that the fire-engine never had to come to our rescue. But the girls I have known who gave way to the habit at least had the good feeling to be ashamed of themselves. According to Lady Jeune, however, the idea that the practice should only be *sub rosa* is vanishing fast, and it is becoming more and more common for ladies to smoke in public. Lady Jeune says that she has seen a young and pretty woman walking along the Strand with a cigarette in her mouth, and another enjoying a cigar with a male companion in Richmond Park; and the testimony of many men is that now women frequently "light up" in a smoking carriage without a shadow of awkwardness or diffidence. So that evidently we women who are sufficiently old-fashioned to like a woman to be a woman will soon be in the minority, and it will not, I suppose, be many years before our schoolgirls as well as our schoolboys will have to be carefully watched by their teachers to prevent injudicious use of the narcotic in their times of recreation.

---

Do you see that a London householder, despairing of obtaining English domestic servants, is anxious to employ Chinamen, and he has advertised in the *Times* for the co-operation of fifty householders, similarly situated, in order to reduce the expenses of agency in China, passages, &c.? His experience is that Chinamen make good servants; and that they are civil, honest, sober, reliable, and invariably good cooks. I expect it is the last item in particular which has so fired the ambition of the Englishman that he is willing to put himself to so much trouble to get Ling Chung or Ah Sin in the place of Sarah Jane or Mary Ann. As to cooking, he is quite correct; the Chinese have natural culinary capacity. But according to the evidence of some relatives of mine, who have lived in China for twenty years, they are not in other respects the treasures English fancy paints them. They do not get drunk with alcohol, but they freely indulge in opium smoking; they are civil enough, but petty pilfering is one of their pet practices; they will work hard, but they want heaps of looking after. I do not think I should like to be one of the first fifty householders to make the Chinese experiment. I would rather pay a little more later on, when the pig-tailed help has become a little more Anglicised, if he is to be introduced at all.

---

The Judge at the Lambeth County Court is, I am afraid, rather hard on the ladies. I dare say it worries him, especially on a very hot day, to have to enquire into cases of dressmakers sued for loss of material through misfits, or dyers summoned for spoiling garments. But I cannot agree with him that the sole reason why a lady, having purchased silk to the value of £5, objects to have it made into a dress which she cannot wear, is because she longs for publicity, and "in her vanity comes to the Court in the hope of getting into print." The suggestion emanating from a man so learned in law, but so unlearned in things feminine, that there should be a court of females to try cases of this kind is, in the circumstances, not unworthy of consideration.

---

Notwithstanding the hot weather, the shops were almost as full as usual last week, and I was considerably amused to observe some of the garments worn. I had been told that a lady at the recent wedding of a noble lord had appeared in a black chiffon gown cut low, back and front, like an evening bodice and filled in with cream-coloured lace up to the neck. But then weddings are always "dressy" gatherings, and I did not fancy the fashion would spread. But many of the fair purchasers in the West End shops the other day seemed to have utilised their ball dresses in much the same way as the lady in the wedding garment. Transparent long sleeves and elbow sleeves were very much *en évidence*, and frequently the bodice, if not unquestionably low, with lace or chiffon arranged to form a sort of covering, was minus any collar, and a ribbon of lace or silk placed round the neck to prevent the decolleté effect being too trying to faces no longer in their first youth. I could not help speculating, should the summer continue to increase in heat as it has done of late, whether ultimately we shall see in real life those pictures by Leech at which we have all laughed, where ladies in muslin frocks with low necks and short sleeves, in lace-work stockings and sandalled shoes, are represented as simpering with the curate, or gently striking the croquet balls. In that case I suppose the modern girl would be "putting" a ball, or smoking with the men under the trees.

### Everybody's Opinion.

[Correspondence on all subjects is invited, but we cannot in any way be responsible for the opinions expressed by our correspondents. No communication can be entertained if the name and address of the correspondent is not given, as a guarantee of good faith but not necessarily for publication, or unless one side of the paper only is written on.]

### FEES IN NORTHERN NURSING INSTITUTIONS.

"Another Lady Superintendent" writes: I am pleased to read "Lady Superintendent's" opinion on ordinary guinea fees. They are certainly inadequate, especially when one considers that nurses are not eligible in any private nursing institution after the age of forty, which leaves a nurse only fifteen years after training to try to save a trifle towards premature old age. My experience also shows that the guinea fee is the chief cause why there is so much difficulty in getting good nurses to work in the northern counties.

### PRIVATE NURSING INSTITUTIONS.

"Matron" writes from The Woodlands, Gildersome, near Leeds: I have read the letters on private nursing institutions, and do not see any advantage to nurses in publishing them. At present it only shows that there are good and bad institutions. If the institutions are as bad as they appear to be, they should be boycotted, and the only way to do that would be to publish the names of them. Then nurses could steer clear of them, and also, as there is always two sides to a story, the particular institute could defend itself, or turn over a new leaf.

### TALKING SHOP.

"A Private Nurse" writes: I hope the letter from "An Old Guy's Nurse" will bear fruit. It is only too true that shop talk by nurses, especially private ones, is very trying, even to fellow workers. What it must be to outsiders is more than I can say. One can scarcely be surprised at people dreading the arrival of a nurse, as I believe many do. The only remedy I can suggest is that superintendents make it a strict rule, even to dismissal, which sounds severe, yet in the end might prove beneficial. Many are splendid women, but the majority might be improved if matrons would be more careful.

### SUGGESTIONS ON THE PAYMENT OF HOSPITAL PATIENTS.

Nurse Copeman, writing from 19, Winchester Road, Oxford, says: As a constant reader of The Hospital, and one who has had many years' experience in hospital and district nursing, may I be permitted to make a suggestion through your columns with regard to the above question. Would it not be a help towards freeing our hospitals from debt, and also preventing their incurring debt, if every patient paid according to his means, and if subscribers' letters were given only to the really necessitous, they, the latter, paying as well sixpence a week? My experience is that it would be quite the exception for any patient or patients' friends not to be able to pay this small sum, and that they would pay it gladly, valuing much more in consequence the benefits received. Of course much more careful investigation would be needed by subscribers, who, I am sorry to say, under the present system, are often grievously deceived in their charitable intentions.

### PARTIAL TRAINING.

Nurse Hyland writes from 17, Belle Vue Terrace, Lancaster, as follows: I am inclined, along with many other fully-qualified nurses, to hold rather strong opinions on the subject of partial *versus* complete training, and I do not agree with the foreign lady's suggestions for such half-and-half measures as a six months' training. Anyone who is willing and capable of helping in times of emergency would be welcomed by the sister—trained help being unavailable. With the three years' system of training so very much upheld throughout the country, how is it possible to advocate a six months' training? I should prefer one with no training whatever to one who had only six months' training. I should then be sure that, whereas the latter, slightly puffed up with her own importance, would be likely to put forward her own views, the former would have to be taught from the beginning. No one who is not in the nursing profession has any idea of how little in connection with real actual nursing one can learn in six months. The first six months of a probationer's life in hospital is spent in having one's mistakes corrected, cleaning brass taps and handles, and in getting generally drilled into order. Some cases may be seen in six months, but how few compared with the multitude of diseases which assail humanity? There is an old adage which says there is no royal road to learning, and certainly there is no such road to nursing. But are we in England so short of well trained nurses that it is necessary to have a "volunteer corps" of untrained women, either for war or epidemic at home or abroad? Surely those who have spent and are spending their lives with the sick are always ready, and willing, aye glad, to put forth their best efforts at the time of their country's need. I think that the real helpers at such a time are those best fitted for the work, namely, the women who have given their lives to the nursing of the sick; not those who have entered the profession perhaps for a change in the monotony of their lives. I hope I am not too severe on ladies, many of whom, I know, enter hospitals for a little training out of a wish to help others and to really do good in the world, but to admit these partially-trained ladies into the profession would certainly lead to confusion. The cooking and dispensing taught in German hospitals is a distinct advantage to the nurse in training, and I often think that at least "sick cookery" should be included in every nurse's training in England, for an ordinary three years' training does not altogether fit one for "private work." There are many things to be learnt on leaving the hospital, "sick cookery" being one of the most important for those wishing to engage in private work. I have read the accout of "Plague Nursing in Poona" by a sister with especial interest, I myself having just returned from plague nursing in Bombay and Dharwar, where I spent the last fourteen months.

### Meath Home of Comfort.

On Tuesday afternoon the annual festival of this institution for epileptic women and children was held at the Home, Godalming. The President, the Duchess of Albany, wrote to the honorary secretary, Mrs. Borrows, regretting her inability to attend, and sent "her best wishes for a good gathering and for the continued prosperity of the Home." The Bishop of Guildford preached the annual sermon—an incident greatly appreciated, as he has never been able to do so before since the establishment of the Home. A very generous response has been made to the special appeal for funds issued last year, more particularly by the Girls' Friendly Society. The members of the society have held concerts and entertainments all over the country, with the result that the £177 borrowed from the very small reserve fund has been replaced. The latest development in the work has been the appointment of a nurse governess, as there are now ten babies to be cared for under nine years of age. Educational work proceeds regularly for all under 18, and some lady patients beyond that age attend improvement classes. There are now 74 patients under treatment.

### Nursing Fund Sunday.

In the neighbourhood of Buntingford it has become a custom to make collections once a year in all the places of worship on behalf of the local nursing fund. This year the amount received at the churches and chapels in five parishes was £8 17s. 3d. It is not large, but the principle is an excellent one, and might with advantage be adopted in other districts where there are no hospitals.

### For Reading to the Sick.

ST. JAMES'S DAY.

"Whosoever will be great amongst you, let him be your minister; and whosoever will be chief among you, let him be your servant."—*St. Matt.* xx. 26-27.

For all Thy Saints, a noble throng,
Who fell by fire and sword,
Who soon were called, or waited long,
We praise Thy name, O Lord;
For him who left his father's side,
Nor lingered by the shore,
When, softer than the weltering tide,
Thy summons glided o'er.
Who stood beside the maiden dead,
Who climbed the mount with Thee,
And saw the glory round Thy head,
One of Thy chosen three;
Who knelt beneath the olive shade,
Who drank Thy cup of pain,
And passed from Herod's flashing blade
To see Thy face again.
—*Hymn for St. James's Day.*

It takes a long time and a sharp discipline to learn that he who would keep his life must first lose it, and that to empty oneself is the sure way to be filled. The heart of man is so constituted that its fulness comes of spending. When we serve—we rule. When we give—we have. When we surrender ourselves—we are victors. We are most ourselves—when we lose sight of ourselves. He is most certain to have his own way and to find pleasure in it who deliberately chooses to resign his preference in favour of others . . . We know not what we are, or might be. As the seed has a tree within it, so men have within them—angels.—*Newman.*

I am confined to one room, I lie on a sick-bed, I am waited on by others; yet my Master does not count me as a cumberer of the ground, an useless member of His household. If I patiently bear what He sends, if I pray to Him and praise Him and trust and love Him, if I receive thankfully what He gives me, and am meek in spirit, and gentle in word, then my gracious Master looks upon me as still doing His will and serving him. And if I can ever speak a word for Him from my sick bed, or tell to any around me how happy I am in His service, or do the least thing to draw any to Him, then, small—and almost nothing—as this service is, yet He condescends to accept it; and, when I ask Him, He will bless what I say or do, and thus be my gracious master still, helping me even in such poor service as this.—*Bourdillon.*

Jesus calls us from the worship
Of the vain world's golden store,
From each idol that would keep us,
Saying "Christian, love Me more."
In our joys and in our sorrows,
Days of toil and hours of ease,
Still He calls, in cares and pleasures,
That we love Him more than these.
Jesus calls us: by Thy mercies,
Saviour, make us hear Thy call,
Give our hearts to Thine obedience,
Serve and love Thee best of all.
—*Hymns A. and M.*

### Death in Our Ranks.

We have to record with much regret the sudden death, on the 19th inst., at Killin, of Nurse Agnes Cumming, Royal Scottish Nursing Institution, Edinburgh. A general favourite both in the Glasgow Royal Infirmary, as staff nurse, and in the Royal Scottish Nursing Institution, she was a most excellent and devoted nurse. Her death was the result of illness contracted in the discharge of duty.

### Notes and Queries.

**The contents of the Editor's Letter-box have now reached such unwieldy proportions that it has become necessary to establish a hard and fast rule regarding Answers to Correspondents. In future, all questions requiring replies will continue to be answered in this column without any fee. If an answer is required by letter, a fee of half-a-crown must be enclosed with the note containing the enquiry. We are always pleased to help our numerous correspondents to the fullest extent, and we can trust them to sympathise in the overwhelming amount of writing which makes the new rules a necessity.**

**Every communication must be accompanied by the writer's name and address, otherwise it will receive no attention.**

*Mentally Afflicted.*

(147) Two nurses holding the medico-psychological certificate, one hospital trained, are thinking of establishing a home for a few mentally-afflicted ladies. They would feel sincerely obliged if you would give them some idea of the probable cost of establishing such a home, and how to set about it.—*A. T.*

A home for mentally-afflicted ladies would be a lunatic asylum, and would come under the provision of the Lunacy Law. We believe that such an institution could not be opened without the consent of the Lunacy Commissioners.

*Employment Abroad.*

(148) 1. Will you kindly inform us if any society or institution exists for employing trained nurses to undertake cases in the South of France during the winter months? 2. Also, where information can be obtained respecting nursing in Johannesburg, its prospects, and the way out?—*A London Nurse.*

1. The Hollond Institute, 1, Tavistock Chambers, Bloomsbury, W., engages nurses for work in the South of France during the winter. As vacancies on the staff occur advertisements appear in the "Mirror." 2. The Emigrants' Information Office, 31, Broadway, Westminster, will give full particulars. Note well the warning issued by the office to young women proceeding to Johannesburg.

*Children's Nurses.*

(149) Kindly tell me if there are not some homes where educated girls are trained specially to become children's nurses, and, if so, the addresses?—*Nurse B.*

Yes. The best known is the Norland Institute for Training Children's Nurses, 29, Holland Park Avenue, Notting Hill, W. Another is the Liverpool Ladies' Sanitary Association, 8, Sandon Terrace, Liverpool. There are also several smaller homes which give this instruction.

*Hygiene.*

(150) Will any of your readers tell me the name of a good work on hygiene?—*E. M.*

"A Manual of Hygiene for Students and Nurses," by John Glaister M.D., D.P.H. (Scientific Press, price 3s. 6d.), is very well spoken of, and is probably just what you want.

*Experience.*

(151) Could you kindly inform me of any place in London (hospital out-patient department, dispensary, or otherwise) where a nurse could attend daily and obtain good practice in surgical dressings and instruction in very minor surgery preparatory to going abroad?—*C. D.*

You might perchance be able to make arrangements with some of the smaller hospitals to which no medical schools are attached. You will find a list of these and of the general and provident dispensaries in "Burdett's Hospitals and Charities." Select those most convenient for you to attend, and apply.

*Colorado.*

(152) I am anxious to go out to Colorado, either Denver or elsewhere. I am a thoroughly trained and experienced nurse. Will you kindly tell me the best way of obtaining work out there?—*Ebor.*

This could only be answered by someone on the spot, but if you apply to Emigrants' Information Office, 31, Broadway, Westminster, you will obtain recent and accurate information as to the general prospects o nurses there.

*Quotation.*

(153) Could you kindly inform me what is the name of, and where to procure, a poem in which the lines

"Laugh, and the world laughs with you;
Weep, and you weep alone,"

occur? I have never seen it in print, and do not know whether it is published in a separate form or in a collection of poems.—*E. L. B.*

We hope some of our readers may be able to supply the desired information.

*Mission Nursing in Africa.*

(154) About six months ago there was an appeal in the "Nursing Mirror" to nurses, on behalf of mission work in Africa. I cannot remember what mission it was. Will you give me any information on the subject, and also inform me to whom application should be made for possible vacancies?—*Superintendent.*

The mission was in connection with an Anglican Sisterhood. Perhaps the Sister in charge will kindly forward it to us for our correspondent.

*Gynæcology.*

(155) Will you kindly recommend me a good gynæcological book for a nurse to have?—*F. C. P.*

We do not know of any reliable handbook of gynæcology specially written for nurses. The standard text-book is "A System of Gynæcology," edited [illegible] T. Clifford Allbutt, M.D., and W. S. Playfair, M.D., published by [illegible]lan.

## Figures and Tables

**Figure f1:**